# Influence of solvent-free extraction of fish oil from catfish (*Clarias magur*) heads using a Taguchi orthogonal array design: A qualitative and quantitative approach

**DOI:** 10.1515/biol-2022-0789

**Published:** 2023-11-23

**Authors:** Jaydeep Dave, Ali Muhammed Moula Ali, Tanaji Kudre, Pikunthong Nukhthamna, Nishant Kumar, Marek Kieliszek, Sri Charan Bindu Bavisetty

**Affiliations:** School of Food-Industry, King Mongkut’s Institute of Technology Ladkrabang, Bangkok 10520, Thailand; Department of Meat and Marine Sciences, Central Food Technological Research Institute, Mysore, Karnataka 570020, India; Department of Food Science and Technology, National Institute of Food Technology Entrepreneurship and Management, Kundli, Sonipat, Haryana, 131028, India; Department of Food Biotechnology and Microbiology, Institute of Food Sciences, Warsaw University of Life Sciences – SGGW, Nowoursynowska 159 C, 02-776, Warsaw, Poland

**Keywords:** catfish waste management, fish oil new resource, solvent-free extraction, polyunsaturated fatty acids, fatty acid analysis, volatile compounds

## Abstract

This study aimed to efficiently utilize catfish heads, enhancing the oil extraction process while improving the cost-effectiveness of fish byproduct management. The study employed the wet rendering method, a solvent-free approach, utilizing a two-factor Taguchi orthogonal array design to identify critical parameters for optimizing oil yield and ensuring high-quality oil attributes. The extraction temperature (80–120°C) and time (5–25 min) were chosen as variables in the wet rendering process. Range analysis identified the extraction time as a more significant (*p* < 0.05) factor for most parameters, including oil yield, oil recovery, acid value, free fatty acids, peroxide value, and thiobarbituric acid reactive substances. The extraction temperature was more significant (*p* < 0.05) for oil color. Consequently, the wet rendering method was optimized, resulting in an extraction temperature of 80°C and an extraction time of 25 min, yielding the highest oil yield. This optimized wet rendering process recovered 6.37 g/100 g of oil with an impressive 54.16% oil recovery rate, demonstrating comparable performance to traditional solvent extraction methods. Moreover, Fourier transfer infrared spectra analysis revealed distinct peaks associated with triacylglycerols and polyunsaturated fatty acids (PUFA). The oil recovered under optimized conditions contained higher levels of PUFA, including oleic acid (189.92 μg/g of oil), linoleic acid (169.92 μg/g of oil), eicosapentaenoic acid (17.41 μg/g of oil), and docosahexaenoic acid (20.82 μg/g of oil). Volatile compound analysis revealed lower levels of secondary oxidation compounds under optimized conditions. This optimized wet rendering method offers practical advantages in terms of cost-efficiency, sustainability, reduced environmental impact, and enhanced oil quality, making it an attractive option for the fish processing industries. Future research possibilities may include the purification of the catfish head oil and its application in the food and pharmaceutical industries.

## 1 Introduction

Catfish is one of the common freshwater fish. In 2021, their global production was estimated to be 5.8 million metric tons annually [1]. The most commonly farmed catfish species for consumption are the channel catfish (*Ictalurus punctatus*) and the African catfish (*Clarias gariepinus*). Another type of catfish, namely *Clarias magur*, is primarily found in South and Southeast Asia and is considered a delicacy in Thailand, Indonesia, Vietnam, India, and Bangladesh [2]. *C. magur* is a relatively new species and has not been fully utilized in aquaculture and food industries to develop products [3]. In addition, only limited research has been done to explore new technologies that enhance the production and management of this fish with its commercialization.

By-product management has changed significantly due to the evolution of aquaculture and fisheries. In recent decades, components like bones, skin, viscera, and trimmings have been transformed into commodities of interest, driven by various scientific studies and commercial importance [4,5]. Generally, catfish processing generates a significant amount of waste, yielding about 40–60% based on the wet weight, including heads, skin, scales, bones, trimmings, viscera, etc. [6]. Nowadays, these by-products are valorized for their bioactive compounds, e.g., bones provide collagen and calcium [7]; skin provides bioactive peptides and collagen [5]; visceral biomass provides enzymes and omega 3-rich oil [8]; and trimmings provide fishmeal. However, the utilization of catfish heads has not yet been adequately researched. Typically, head waste is either converted to low-valued animal feed and fertilizer or discarded as waste, which can lead to environmental hazards [9].

Generally, the fish head is known to contain a significant amount of nutritional compounds, such as proteins, lipids (rich in saturated fatty acids, monounsaturated fatty acids [MUFA], and polyunsaturated fatty acids [PUFA]), pigments, vitamins, and minerals, which are from various parts, including the brain, eyeballs, gills, and cheeks [10]. Catfish head oil has been reported to contain oleic acid, linoleic acid, alpha-linolenic acid (ALA), eicosapentaenoic acid (EPA), and docosahexaenoic acid (DHA), which have been linked to numerous health benefits, including improved heart and brain health, healthy skin maintenance, reduced inflammation, and lowered cholesterol and triglycerides [8,11]. Catfish heads are often overlooked in the fish processing industry due to a lack of awareness about their potential uses and cultural preferences for fillets [6]. Limited culinary knowledge and aesthetic concerns also contribute to their underutilization [12]. However, catfish heads are nutrient-rich and can offer health benefits [3]. With the focus on reducing seafood waste and sustainable practices, finding effective ways to utilize catfish heads is important. Extracting oil from fish heads can be challenging due to their tough structure and the presence of impurities like proteins [13]. Overall, the research gap lies in the need to explore and develop innovative and sustainable methods for the efficient extraction and utilization of catfish head oil, addressing both environmental and economic considerations.

Typically, recovery of fish oil has been made using various chemical solvents, enzymatic hydrolysis, supercritical fluid extraction, wet rendering, and cold pressing [14]. However, some of these methods are linked to several drawbacks, e.g., chemical solvent extraction still contains traces of hazardous chemicals, and the extraction process is not eco-friendly. Cold press renders a very low oil yield. Enzymatic and supercritical fluid extraction is associated with higher capital expenditures and, hence, is not cost-effective. Due to this reason, the present research is focused on solvent-free, eco-friendly, and cost-effective methods for recovering fish oil [15,16]. In this regard, wet rendering is gaining interest, which involves cooking fish biomass in water or steam to break the complex tissue and release the oil in free form. This oil is easily separated from the water and other fish solids. Besides, this method has several advantages over other methods, such as considerable oil yields, lower operational or capital costs, no involvement of toxic chemicals, etc. [10].

The objective of this work is to address the existing research gap by introducing the wet rendering method as an effective way of extracting valuable oil from catfish heads. This approach aims to enhance the efficient and sustainable usage of this underutilized resource. Nazir et al. [13] observed that cooking time and temperature can affect the yield and quality of fish oil during wet rendering of tuna heads. The optimization of factors using experimental design is essential in the context of wet rendering for maximizing the oil yield and preservation of PUFA. The Taguchi orthogonal array design enables efficient exploration of multiple factors, identification of critical variables, and resource savings, and provides information about the contribution of individual treatment factors [17]. This information would be specifically valuable for scaling up the extraction, considering less number of factors critically affecting the quality of extracted oil. The efficient extraction of fish oil from catfish heads not only reduces waste but also creates new avenues for product development across multiple industries including food, pharmaceuticals, and cosmetics. It also promotes sustainability and economic growth.

## 2 Materials and methods

### 2.1 Chemicals

Thiobarbituric acid (TBA), trichloroacetic acid, ammonium thiocyanate, ferrous chloride, and sodium hydroxide (NaOH) were purchased from Merck (Darmstadt, Germany). Solvents, including chloroform, methanol, and hexane, were obtained from Lab-Scan (Bangkok, Thailand). Supelco^®^ 37 component fatty acid methyl ester (FAME) mix, 1,1,3,3-tetramethoxypropane, and ammonium thiocyanate were procured from Sigma-Aldrich (St. Louis, MO, USA). Cumene hydroperoxide and 2-TBA were obtained from Fluka Co. (Buchs, St. Gallen, Switzerland). The other chemicals were obtained from Merck (Darmstadt, Germany). All the chemicals used were of analytical grade.

### 2.2 Raw materials

Freshly available *C. magur* heads were procured from the Huatake fish market located at Ladkrabang, Bangkok, Thailand. Approximately, 2–3 kg of heads were packed in polyethylene bags (thickness, 63.50 microns, placed in a polystyrene container containing ice, and transported to the Faculty of Food-Industry, KMITL. Upon arrival, the heads were chopped into pieces and minced using a meat grinding machine (JYR-120, Bangkok, Thailand), and the minced samples were used for further experiments.

### 2.3 Proximate composition

The proximate composition was determined in terms of moisture, proteins, fats, and ash using the AOAC method (930.15, 923.03, 920.39, and 923.01), respectively [18].

### 2.4 Experimental design

The Taguchi orthogonal array design was employed, to identify the most significant contributing factor from the commonly used two factors, with the assumption that there was no interaction between them. This design provided a simple and structured methodology to minimize the number of treatment combinations when dealing with multiple factors. The Taguchi design employed two parameters to identify the most important factor, which had a significant impact. The first parameter, *K*_*ij*_, was calculated as the average value of the measured functional parameter at each level (*j* = 1, 2, 3) of each factor (*i* = A, B), which was calculated using the following equation:

(1) \[{K}_{{ij}}=1/{N}_{i}\mathop{\sum }\limits_{u=1}^{{N}_{i}}{y}_{i,j,u},]\]

where *N*_*i*_ represents the number of trials for each factor; and *y*_*i*,*j*_ are the measured responses of factor *i* at level *j*. The second parameter, the difference between the highest and lowest values of *K*_*ij*_, is represented as *R*_*i*_. The most contributing factor for extraction was chosen considering both the rank of *R*_*i*_ values for each factor and the factors showing significance (*p* < 0.05) by analysis of variance.

The wet rendering conditions (a combination of the extraction temperature and time) were optimized based on the outcomes of the Taguchi design in order to get the maximum oil yield. To check the reliability of the optimized conditions, a validation step was carried out by considering the influence of most contributing factors on the responses.

### 2.5 Extraction of oil from catfish heads

A minced sample (100 g) was used for oil extraction by the wet rendering method (solvent-free extraction). The extraction was carried out using two variables at three levels, i.e., cooking temperatures (80, 100, and 120°C) and cooking times (5, 15, and 25 min). The ratio of the minced sample to water was kept constant at 1:0.5 (w/v) (values were taken based on the previous optimization conditions, data not shown). The two factors at three levels for extraction were optimized using a three-level Taguchi orthogonal array design, as shown in Table 1. After extraction, the samples were allowed to cool at room temperature and filtered using a muslin cloth (to remove larger particles and cell debris), followed by filtration using Whatman no 1 filter paper (to remove the cell debris and fine particles). Then, the samples were centrifuged at 2,500×*g* for 15 min to separate the oil and aqueous phases with some pellets. The oil was separated using a separating flask and the recovered oil was subjected to analysis. The excess oil was stored in an amber bottle, head space flushed with nitrogen gas, and stored at −20°C (not more than a month). The oils extracted using the solvent method, i.e., chloroform:methanol (2:1, v-v) and hexane, were considered as controls [19].

**Table 1:** Runs showing two factors for oil extraction using the wet rendering method obtained via a Taguchi orthogonal array design

Run	Factor A: Temperature (°C)	Factor B: Time (min)
1	80	5
2	80	15
3	80	25
4	100	5
5	100	15
6	100	25
7	120	5
8	120	15
9	120	25

### 2.6 Extraction yield

The obtained oil was analyzed for extraction yield, expressed in terms of g/100 g (wet weight basis), and was calculated using the following formula:

(2) \[{\mathrm{Yield}}({\mathrm{g}}\left/100{\mathrm{g}})=\frac{{\mathrm{Weight\; of\; the\; obtained\; oil}}({\mathrm{g}})}{{\mathrm{Weight\; of\; sample\; taken\; for\; extraction}}({\mathrm{g}})}\times 100.]\]

The oil recovery was calculated using the following formula and expressed in percentage (%):

(3) \[{\mathrm{Oil\; recovery}}(\left \% )=\frac{{\mathrm{Weight\; of\; obtained\; oil}}\left({\mathrm{g}})}{{\mathrm{Total\; oil\; present\; in\; the\; sample}}({\mathrm{g}})}\times 100.]\]

### 2.7 Quality characteristics

#### 2.7.1 Color

The color was determined using a HunterLab colorimeter (LabScan^®^ XE, Reston, VA, USA) and reported in the CIE color system, where *L* ^*^ = lightness/brightness*, a* ^*^ = redness/greenness, *b* ^*^ = yellowness/blueness, and Δ*E* ^*^ is the color difference. The corresponding values of a white standard were *L* ^*^ = 93.71, *a* ^*^ = −1.21, and *b* ^*^ = 0.48.

#### 2.7.2 Acid value and free fatty acids (FFA)

The acid value and FFA were analyzed as described by Chaijan et al. [20] with slight modifications. The sample (1 g) was dissolved in ten volumes of hexane (w:v) containing two drops of phenolphthalein indicator. The mixture was titrated against 0.02 N potassium hydroxide (KOH) solution until a pink color was achieved. The acid value was expressed as mg KOH consumed to neutralize the free acids in 1 g of oil and was calculated using the following formula:

(4) \[{\mathrm{Acid\; value}}=\frac{56.1\times N\left\times ({V}_{{\mathrm{s}}}\left-{V}_{{\mathrm{b}}})}{W},]\]

where *N* is the normality of KOH, *V*_s_ is the volume of KOH required for the sample, *V*_b_ is the volume of KOH required for the blank, and *W* is the weight of the sample (in g).

The FFA in oil was expressed as % oleic acid, calculated using the following formula:

(5) \[{\mathrm{FFA}}(\left \% )=\frac{{\mathrm{Acid\; value}}\times {\mathrm{Molecular\; weight\; of\; oleic\; acid}}\times 10}{{\mathrm{Molecular\; weight\; of\; KOH}}\times 1,000}]\]

### 2.8 Oxidative stability

#### 2.8.1 Peroxide value (PV)

The PV was determined using the spectrophotometric method described by Bruno et al. [21], and 20 mM ferric thiocyanate was used as a reducing agent. A standard curve was plotted using cumene hydroperoxide at concentrations in the range of 0–50 ppm. The PV was expressed in mg cumene equivalents/kg of oil sample.

#### 2.8.2 TBA reactive substances (TBARS)

The analysis of TBARS was carried out as described by Bruno et al. [21] with slight modifications. The oil (10 mg) was mixed with 2.5 mL of a TBA reagent and incubated at 95°C for 10 min. The reaction was stopped by cooling the test tubes under running tap water and then centrifuged at 3,600×*g* for 20 min at 25°C. The upper layer was collected, and absorbance was recorded at 532 nm. The TBARS content was measured using a standard curve plotted with 1,1,3,3-tetra ethoxy propane and expressed as mg of malonaldehyde (MDA)/kg oil.

### 2.9 Characterization

Four oil samples were selected for characterization: (1) oil sample (Run 3) with the highest yield and lower oxidation (optimized condition), and (2) oil sample (Run 7) with high oxidation with lower yield was selected as a negative control, (3) chloroform:methanol (2:1, v/v) as a positive control, and (4) hexane as a positive control.

#### 2.9.1 Fourier transfer infrared (FTIR) spectroscopy

FTIR analysis was carried out using the ATR-FTIR model Equinox 55 (Bruker, Ettlingen, Germany) in the range of 400–4,000 cm^−1^ with a resolution rate of 4 cm^−1^. The spectra were interpreted as described by Ali et al. [19].

#### 2.9.2 Fatty acid composition

FAMEs were prepared as described by Muhammed et al. [22] using 2 N methanolic HCl and 2 N methanolic NaOH solutions. The derivatized FAMEs (1 μl) were injected into a gas chromatography (7890B series; Agilent Technologies, Santa Clara, CA, USA) equipped with a flame ionization detector and an HP 88 capillary column (J&W Scientific Column from Agilent Technologies). The GC conditions were set according to the instructions provided by the manufacturer. The peaks were identified based on the matching retention time with the Supelco^®^ external standard (37 Component FAME Mix). The identified peaks were integrated and calibrated against the standard concentration curve using Open LAB CDS software (Chem Station edition; Agilent Technologies, Santa Clara, CA, USA). From the fatty acid composition data, the chemometric analysis was performed to characterize the fish oil [23].

#### 2.9.3 Volatile compounds

Volatile compounds were measured using a gas chromatography-mass spectrometer (HP 5890 series II; Hewlett Packard, Atlanta, GA, USA) equipped with an HP 5972 quadrupole mass detector, as outlined by Ali et al. [24]. Ionization was performed at 250°C, in an electron ionization mode. The spectra were obtained at an ionization energy of 70 eV. The acquisition was monitored at a scanning rate of 0.22 s/scan in the range of 25–500 amu. The ChemStation Library (Library No. Wiley 275. L) was used to identify the volatile compounds. The volatile compounds are expressed in abundance based on the peak area.

### 2.10 Statistical analysis

The experiments were performed in triplicates, and the data were presented in mean ± SD. Duncan’s multiple range test was used to enumerate the significant difference between mean values. Range analysis was carried out using SPSS (version 10.0.1.0, Stat-Ease) to check the effect of individual variables and to determine the optimum level of different variables. The average response (*P*_*ij*_) for each variable (*i* = 1–2) was calculated at each level (*j* = 1–3). The difference between the highest and lowest values of *P*_*ij*_ is represented as *R*_*j*_ and was calculated to determine the most contributing variable based on rank (*R*_*j*_) values of each variable on quality attributes of the oil.

## 3 Results and discussion

The proximate composition of *C*. *magur* head contained 65.78% moisture, 15.05% proteins, 11.76% fat, and 4.91% ash (wet weight basis). Based on the data from previous literature, the fat content from catfish heads ranges from 7.22 to 13.51%, based on the wet weight. These variations could depend on the species, habitat, diet, and physiological conditions [25].

### 3.1 Effect of most contributing extraction variable based on the range analysis

In the present study, a significant (*p* < 0.05) effect of independent variables was observed on the extraction yield, quality characteristics, and oxidative stability of oils recovered through wet rendering.

#### 3.1.1 Extraction yield

Based on the range analysis experiments, the oil yield ranged from 2.48 to 6.37 g/100 g, and oil recovery ranged from 21.08 to 54.16%, as shown in Table 2. The results showed that time was the most dominant parameter; with an increase in time, there was a significant increase in the extraction yield and recovery, as presented in Table 3. On the other hand, the temperature did not significantly affect the extraction. Kudre et al. [26] also observed that a longer extraction period effectively increased the yield. Some oil compounds may be tightly bound within the fish matrix, especially if they are trapped within cellular structures or associated with proteins. Longer extraction times provide more opportunities for the solvent to break down these barriers and release the oil [27].

**Table 2:** Yield, recovery, color, and quality parameters of catfish head oils recovered using the wet rendering method

Run	Oil yield^a^	Oil recovery (%)	*L* ^*^	*a* ^*^	*b* ^*^	Δ*E* ^*^	Acid value^b^	FFA^c^	PV^d^	TBARS^e^
1	2.48 ± 0.34^a^	21.08 ± 1.70^a^	78.17 ± 0.11^c^	−1.90 ± 0.02^c^	40.99 ± 0.05^e^	43.45 ± 0.13^e^	11.11 ± 0.12^a^	0.56 ± 0.02^b^	21.29 ± 0.32^c^	0.32 ± 0.03^a^
2	4.21 ± 0.50^c^	35.79 ± 1.21^c^	78.22 ± 0.15^c^	−1.91 ± 0.01^c^	40.98 ± 0.03^e^	43.42 ± 0.24^e^	24.82 ± 0.34^e^	1.40 ± 0.03^e^	39.52 ± 0.29^g^	0.39 ± 0.02^b^
3	6.37 ± 0.63^f^	54.16 ± 0.89^f^	78.19 ± 0.08^c^	−1.90 ± 0.03^c^	40.97 ± 0.08^e^	43.43 ± 0.15^e^	28.13 ± 0.62^f^	1.62 ± 0.06^f^	28.34 ± 0.33^e^	0.51 ± 0.03^d^
4	3.26 ± 0.28^b^	27.72 ± 1.21^b^	77.14 ± 0.09^b^	−1.89 ± 0.02^b^	38.92 ± 0.02^e^	43.36 ± 0.28^e^	18.16 ± 0.31^b^	0.91 ± 0.04^c^	29.21 ± 0.28^e^	0.34 ± 0.02^a^
5	5.16 ± 0.26^d^	43.87 ± 0.69^e^	77.29 ± 0.03^b^	−1.89 ± 0.02^b^	38.61 ± 0.02^d^	43.05 ± 0.31^d^	23.61 ± 0.39^d^	1.19 ± 0.02^g^	34.37 ± 0.41^f^	0.45 ± 0.07^c^
6	5.69 ± 0.59^e^	52.63 ± 1.05^f^	77.41 ± 0.10^b^	−1.85 ± 0.01^b^	39.44 ± 0.03^c^	42.85 ± 0.29^c^	44.27 ± 0.42^h^	2.23 ± 0.05^i^	23.64 ± 0.68^d^	0.54 ± 0.07^e^
7	4.89 ± 0.51^c^	41.58 ± 1.01^d^	76.69 ± 0.17^a^	−1.80 ± 0.01^a^	37.45 ± 0.01^c^	42.76 ± 0.17^b^	22.18 ± 0.19^c^	1.12 ± 0.07^d^	34.67 ± 0.29^f^	0.46 ± 0.05^c^
8	5.18 ± 0.58^d^	44.30 ± 0.87^e^	76.81 ± 0.11^a^	−1.77 ± 0.04^a^	37.31 ± 0.02^b^	42.59 ± 0.38^a^	39.88 ± 0.21^g^	2.01 ± 0.02^h^	21.84 ± 0.54^c^	0.58 ± 0.06^f^
9	5.21 ± 0.46^d^	44.04 ± 0.64^e^	76.89 ± 0.07^a^	−1.78 ± 0.02^a^	37.22 ± 0.05^a^	42.47 ± 0.24^a^	48.23 ± 0.49^i^	2.43 ± 0.04^j^	14.12 ± 0.45^b^	0.64 ± 0.04^g^
A	8.29 ± 0.34	70.05 ± 0.74	76.28 ± 0.04	−1.88 ± 0.02	17.86 ± 0.03	24.12 ± 0.13	9.34 ± 0.21	0.47 ± 0.02	7.78 ± 0.56	0.12 ± 0.02
B	7.64 ± 0.27	64.55 ± 0.62	77.11 ± 0.10	−1.86 ± 0.03	18.35 ± 0.01	23.92 ± 0.05	8.21 ± 0.18	0.41 ± 0.01	6.68 ± 0.39	0.11 ± 0.01

Solvent extraction methods i.e., hexane and chloroform:methanol (2:1, v/v) were positive controls. Values are given as mean ± SD (*n* = 3). The same lowercase letters of each measured parameter indicated a significant difference (*p* < 0.05) during wet rendering.

^a^Oil yield is expressed in terms of g/100 g, based on the wet weight; ^b^acid value is expressed in terms of mg KOH/g of oil; ^c^FFA is expressed in terms of % oleic acid; ^d^PV is expressed in terms of mg of cumene hydroperoxide equivalent/kg of oil; ^e^TBARS is expressed in terms of mg of MDA Eq/kg of oil.

Runs 1–9: oil recovered using wet rendering.

A: Oil extracted by chloroform:methanol (2:1, v/v); B: oil extracted by hexane.

**Table 3:** Range analysis from factorial design for response variables against the yield, recovery, color, and quality parameters of catfish head oils

Factor	Levels	Values	Oil yield^a^	Oil recovery (%)	*L* ^*^	*a* ^*^	*b* ^*^	Δ*E* ^*^	Acid value^b^	FFA^c^	PV^d^	TBARS^e^
A	80	*K*_A1_	4.35^a^	37.01^a^	78.19^a^	−1.90^c^	40.98^c^	43.40^c^	43.68^a^	2.20^a^	29.71^c^	0.40^a^
	100	*K*_A2_	4.70^b^	41.40^b^	78.31^b^	−1.87^b^	40.65^b^	43.08^b^	52.01^b^	2.61^b^	29.07^b^	0.44^b^
	120	*K*_A3_	5.09^c^	43.30^c^	78.79^c^	−1.78^a^	40.32^a^	42.60^a^	56.76^c^	2.85^c^	23.54^a^	0.56^c^
		*R*_A_	0.74	6.29	0.48	−0.12	0.66	0.80	13.08	0.65	6.17	0.16
B	5	*K*_B1_	3.54^a^	30.12^a^	78.36^a^	−1.86^a^	40.78^c^	43.19^c^	37.15^a^	1.87^a^	28.39^b^	0.37^a^
	15	*K*_B2_	4.86^b^	41.32^b^	78.44^b^	−1.85^a^	40.63^b^	43.02^b^	53.77^b^	2.70^b^	31.91^c^	0.47^b^
	25	*K*_B3_	5.74^c^	50.27^c^	78.49^b^	−1.84^a^	40.54^a^	42.91^a^	61.54^c^	3.09^c^	22.03^a^	0.56^c^
		*R*_B_	2.20	20.15	0.13	−0.02	0.24	0.28	24.39	1.22	9.88	0.19
Rank			B^*^ > A	B^*^ > A	A^*^ > B	A^*^ > B	A^*^ > B	A^*^ > B	B^*^ > A	B^*^ > A	B^*^ > A	B^*^ > A
Major contributing factor			B	B	A	A	A	A	B	B	B	B

A and B represent s temperature (°C) and time (min), respectively.

^a^Oil yield is expressed in terms of g/100 g, based on the wet weight; ^b^acid value is expressed in terms of mg KOH/g of oil; ^c^FFA is expressed in terms of % oleic acid; ^d^PV is expressed in terms of mg cumene hydroperoxide equivalent/kg of oil; ^e^TBARS is expressed as in terms of mg MDAEq/kg of oil.

*K*_*ij*_ were the average values of each measured parameter from nine treatment sets in level *j* (*j* = 1, 2, 3) of each factor *i* (*i* = A or B).

The same lowercase letters above *K*_*ij*_ of each measured parameter indicated a significant difference (*p* < 0.05).

*R*_A_ and *R*_B_ were the differences between the highest and lowest values of *K*_*ij*_ within the same factor *i*.

Rank was based on the largest to smallest order of *R*_A_ and *R*_B_ values; the factor with an asterisk meant significance detected. The two most contributing factors were selected based on the significance and rank order of *R*_*i*_.

#### 3.1.2 Quality characteristics

The color of fish oil is crucial for quality control in the food and cosmetic industries, where the oil color can significantly affect the final product’s appearance and consumer acceptability [28]. Range analysis showed that the extraction temperature was the most significant (*p* < 0.05) parameter affecting the color values (Table 3). In the wet rendering, the *L* ^*^ (lightness) values varied from 76.89 to 78.17. The increase in temperature resulted in a decrease in lightness. The key factor contributing to the color of fish oil is the presence of pigments, including carotenoids, which are responsible for the oil’s lightness or coloration [29]. When the extraction temperature increases, it may trigger thermal degradation of these pigments. The breakdown of these pigments due to increased temperature ultimately leads to a darker and less light oil. For this, several researchers have proposed different mechanisms. For example, Lanka and Jayewardenepura [30] reported that higher temperatures can affect the interactions between proteins and lipids present in the oil. Changes in these interactions can influence the dispersion of pigments and other color-contributing compounds in the oil, potentially leading to a decrease in lightness. In another study, Grebenteuch et al. [31] reported that high temperatures could polymerize browning substances by condensation and dehydration of the secondary products from lipid oxidation during the wet rendering method. Furthermore, *a* ^*^ values (−1.78 to −1.91) and *b* ^*^ values (37.22–40.99) were also significantly (*p* < 0.05) decreased with an increase in the temperature. Higher temperatures can promote the Maillard reaction, a complex chemical reaction between amino acids (from proteins) and reducing sugars [32]. This reaction often results in browning and the formation of melanoidin compounds, which can impart a darker color to the oil. Lanka and Jayewardenepura [30] reported that oxidation reactions could occur at high temperatures, forming colored compounds that contribute to a darker color. Additionally, Grebenteuch et al. [31] reported that high temperatures could increase the thermal degradation of pigments that are naturally present in the oil, further reducing its color values.

Acid value and FFA are important parameters for assessing the quality and freshness of fish oils [33]. Elevated acid values can indicate oil degradation, rancidity, or hydrolysis. During wet rendering, the range analysis demonstrated that the acid value and FFA were most significantly (*p <* 0.05) affected by the extraction time (Table 3). It was observed that with an increase in the extraction time, there was a gradual increase in the acid value and FFA. This could be possibly due to increased hydrolysis of fat and oil to release FFA [13]. Longer extraction time during wet rendering means prolonged exposure to heat and moisture. During this time, water can catalyze hydrolytic reactions known as lipolysis. In lipolysis, water molecules cleave the ester bonds in triglycerides, breaking them down into glycerol and individual fatty acids [10]. This process releases FFA into the oil phase. Fish and other biological tissues contain enzymes, such as lipases, that can catalyze the hydrolysis of triglycerides into FFA [34]. These enzymes become more active as the extraction time prolongs, leading to greater hydrolysis. Zhang et al. [35] observed that the moisture content of the fish tissue can influence hydrolysis. Increased extraction time can allow for more interaction between moisture and lipids, promoting hydrolysis. Optimization of extraction time can lead to cost efficiency by reducing the energy and time required for the wet rendering process. Efficient production processes can translate into lower production costs.

#### 3.1.3 Oxidative stability

PV is a critical parameter for assessing the freshness and oxidative stability of fish oils. Higher PV values indicate a higher level of primary oxidation products, which can contribute to off-flavors and reduced shelf life [36]. Monitoring PV helps in assessing the oil’s quality and suitability for various applications. Range analysis showed that the extraction time was the most significant (*p <* 0.05) parameter affecting the PV of oil, recovered through wet rendering (Table 3). During wet rendering, from 5 to 15 min, a slight increase in PV was observed, and from 15 to 25 min, it resulted in decreased PV (Table 2). This can be supported by the hydrolysis of ester bonds of triglyceride occurring more at prolonged extraction time, which elevates the level of FFA, which reacts with oxygen and generates hydroperoxides. In the initial stages of wet rendering (5–15 min), there may be an initiation of lipid oxidation. Lipid oxidation is a complex chain reaction that begins with the formation of hydroperoxides, which contribute to the PV [37]. This may lead to a slight increase in PV during this phase as these hydroperoxides are formed. As the wet rendering process continues beyond 15 min, various factors can contribute to the consumption of peroxides. As the reaction progresses, the rate of peroxide decomposition may become more dominant, resulting in a decrease in PV. Lu [38] also observed decreased PV during the cooking of mackerel fillets for 30 min, which could be due to prolonged cooking time where the initial hydroperoxides might decompose into various volatile and non-volatile products. The range analysis showed that time was the most significant parameter (*p <* 0.05) affecting the TBARS, during wet rendering (Table 3). With an increase in time, a gradual increase in TBARS was observed, irrespective of the temperature. The prolonged cooking may convert the primary oxidation products into secondary oxidation products like aldehydes, i.e., MDA. Similar results were observed by Sajib et al. [39], where the TBARS increased during the prolonged cooking of herring fish fillets for 30 min to extract the fish oil.

### 3.2 Optimization and validation of wet rendering process

**Figure 1:**
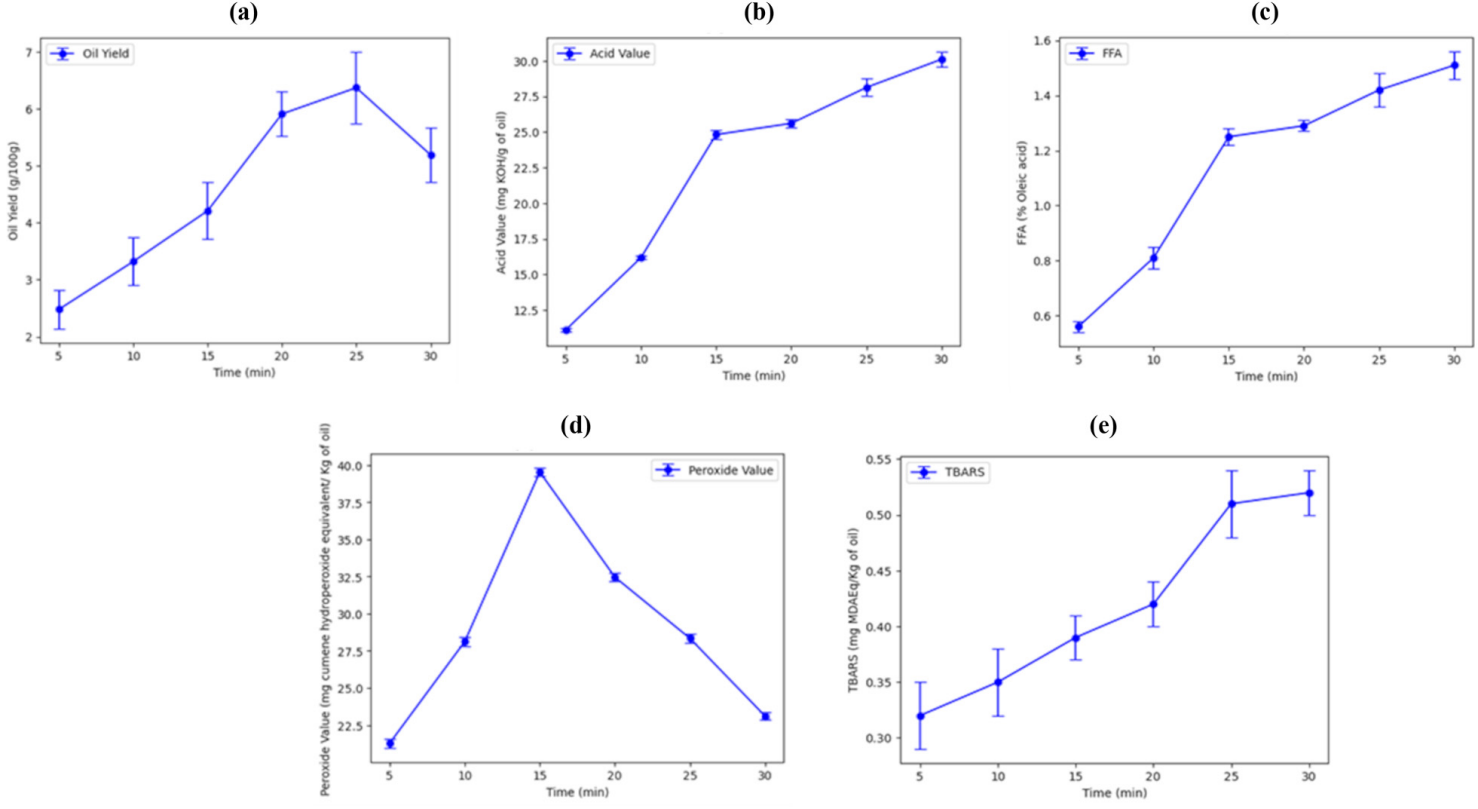
The effect of time (most contributing factor) on the (a) oil yield, (b) acid value, (c) FFA, (d) PV, and (e) TBARS of catfish head oil recovered using wet rendering at 80°C.

Based on previous range analysis observations, it was determined that time had the most significant impact on parameters, such as oil yield, acid value, FFA, PV, and TBARS. Consequently, the extraction conditions for wet rendering were optimized based on maximizing the oil yield. The optimized conditions for wet rendering, which resulted in the highest yield, were an extraction temperature of 80°C and an extraction time of 25 min (as determined in run 3). This particular run yielded an oil yield of 6.37 g/100 g. To validate these results and further examine the influence of time, a wet rendering experiment was conducted at a constant temperature of 80°C (Figure 1).

The time was varied from 5 to 30 min to assess its effect on the parameters of interest. The results obtained from the experiment indicated a significant (*p* < 0.05) increase in the oil yield until 25 min. This increase can be attributed to the greater breakdown of adipose tissues over time, leading to the release of more oil [26]. As discussed earlier, the extended extraction time allowed for a more thorough extraction of oil from the raw material. However, beyond 25 min, there was a noticeable decrease in the oil yield. In this context, the prolonged extraction time likely led to a more extensive denaturation of proteins, exposing their hydrophobic domains [19]. This exposure could result in increased hydrophobic interactions between proteins and lipids. These enhanced protein–lipid interactions could have several effects. One significant outcome is the retention of oil within the protein matrix. When lipids bind more strongly to denatured proteins, they become less available for extraction, reducing the overall oil yield. Additionally, the formation of protein–lipid complexes can alter the solubility of both components within the matrix [40]. Similar trends were observed for the PV and TBARS. With an increase in the extraction time, there was a gradual increase in both PV and TBARS. This phenomenon can be attributed to the increased hydrolysis of fats and oils, leading to the release of FFA [13]. As the extraction time prolongs, there is more opportunity for unsaturated fatty acids to react with oxygen, leading to the formation of hydroperoxides (PV) and secondary oxidation products (TBARS) [41]. Overall, the significant influence of extraction time on the oil yield and quality parameters holds practical importance in ensuring product quality, cost efficiency, shelf life, environmental impact, and process optimization, in the fish processing industry.

#### 3.2.1 Effect of extraction methods on the oil yield, quality characteristics, and oxidative stability

The optimized condition (Run 3) from the Taguchi orthogonal array design recovered 6.37 g/100 g of oil with 54.16% of oil recovery. When compared to the traditional solvent extraction method (chloroform:methanol (2:1, v/v), the oil yield and oil recovery under optimized conditions were 0.76- and 0.77-fold, respectively, lower (Table 2); the values were comparable and not too low with the prospect of any extraction method. Solvent extraction has the advantage of being highly selective for oil, effectively separating it from other components [13]. In wet rendering, the extraction process is less selective and may result in the retention of some oil within the solid residues. The wet rendering method, despite slightly lower oil yield and recovery, offers sustainability advantages. It is a solvent-free, environmentally friendly approach that reduces the use of hazardous chemicals, aligning with the growing demand for sustainable and eco-friendly extraction processes [42]. Furthermore, the acid value of the optimized wet rendering method was 28.13 mg KOH/g oil, which was 3.44-fold higher than that with chloroform:methanol (2:1, v/v) and 3.82-fold higher than with hexane (*p <* 0.05). A higher acid value in the wet rendering process indicates that an increased amount of FFA is hydrolyzed from the glycerol backbone at the ester bond [30]. The FFA content was also observed higher in the wet rendering method, which was 5.17-fold higher than that with chloroform:methanol (2:1, v/v) and 5.92-fold higher than with hexane (*p <* 0.05). The acid value and FFA content of the oil extracted through optimized wet rendering were found within the maximum limits specified by the Codex standard for fish oil i.e., acid value ≤ 30 mg KOH/g oil and FFA ≤ 15% [43]. This demonstrates that the oil obtained through wet rendering meets the regulatory requirements for quality and safety. This compliance is crucial for ensuring that the oil can be legally marketed and used in various food and pharmaceutical applications.

The PV under optimized conditions was 28.34 mg cumene hydroperoxide equivalent/kg oil, which was found to be 3.64-fold higher than that with chloroform:methanol (2:1, v/v) and 4.24-fold higher than the oil recovered with hexane (*p <* 0.05). During the wet rendering of tuna eyeballs, Pudtikajorn and Benjakul [10] also observed higher PV due to the thermal breakdown of lipid molecules, promoting the release of reactive oxygen species. Similarly, the TBARS of oil extracted using the wet rendering method was higher, which was 4.25-fold higher than with chloroform:methanol (2:1, v/v) and 4.63-fold higher than the oil recovered with hexane (*p <* 0.05) under optimal conditions. The high-value TBARS might be due to higher thermal degradation of fatty acids during wet rendering, resulting in more secondary oxidation products like MDAs [44]. These results were in line with that of volatile compounds detected in the oil samples.

### 3.3 Characterization

#### 3.3.1 FTIR spectra

**Figure 2:**
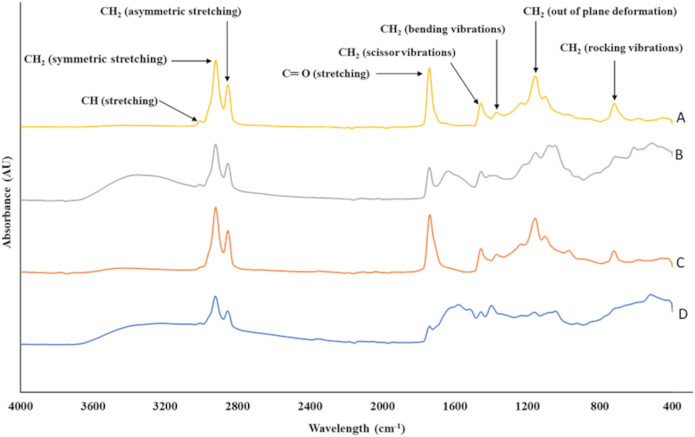
FTIR spectroscopy of catfish head oils recovered using different methods: (a) hexane, (b) chloroform:methanol (2:1, v/v), (c) optimized conditions (extraction temperature = 80°C, extraction time = 25 min), (d) Run 7 (extraction temperature = 120°C, extraction time = 5 min).

Figure 2 illustrates the FTIR spectra of oil samples obtained from the wet rendering and solvent extraction methods. The functional group region of the spectra displayed five distinct peaks. The peak at 3,010 cm^−1^, which indicates the C–H stretching vibration of *cis*-double bonds in fatty acids [45], was observed at low intensity in the solvent-extracted samples but was absent in the oil obtained from the wet rendering method. This discrepancy could be attributed to the thermal degradation effect, which reduces the number of double bonds in fatty acids during the wet rendering process [46]. Additionally, two major peaks were observed at wavenumbers 2,924 and 2,852 cm^−1^, which correspond to the symmetric and asymmetric stretching vibrations of CH_2_, respectively [45]. These peaks are indicative of the fatty acid chain length. Comparing these two peaks, it was noted that the amplitude of the oil sample recovered with chloroform:methanol (2:1, v/v) and Run 7 (low oil quality) was lower than that of the oil samples obtained with hexane under optimized conditions. This reduction in amplitude may be due to thermal degradation, which breaks down the long fatty acid chains [47]. Furthermore, a significant peak at wavenumber 1,742 cm^−1^ corresponding to the C═O stretching vibration of the ester group in triacylglycerols was identified. The intensity of this peak for oil obtained with hexane under optimized conditions was higher than that of the other samples, suggesting a higher presence of ester bonds in triacylglycerols. Conversely, the oil sample from Run 7 showed very low peak intensity, indicating that the ester bond of triacylglycerols had been cleaved by hot water during the wet rendering process [48]. The symmetric CH_2_ stretching vibration, associated with the C═O stretching in triacylglycerols, could provide information about the structural changes and interactions within lipoproteins [45]. Finally, the presence of unsaturated fatty acid chains in the oil samples was indicated by a peak at wavenumber 1,451 cm^−1^, corresponding to the CH_2_ scissor vibration. The oil sample from Run 7 did not exhibit this particular peak, whereas the other samples did. Among all the oil samples, the oil obtained with hexane had the highest amplitude, followed by the oil obtained under optimized conditions. Also, these findings were in agreement with the fatty acid compositions of the oil described in Table 4. The oil obtained by solvent extraction (Samples A and B) displayed a higher number of average double bonds (1.72), while the oil obtained under optimized conditions exhibited intermediate levels (1.70). In contrast, Run 7 showed the lowest number of average double bonds (1.53).

**Table 4:** Change in the fatty acid composition of catfish head oils recovered using the wet rendering method compared to the two solvent extraction methods

Fatty acids	A	B	Optimal conditions*	Run 7**
	(μg/g of oil)			
C14	22.62 ± 0.03^a^	22.61 ± 0.03^a^	21.31 ± 0.01^b^	19.21 ± 0.02^c^
C14:1	2.31 ± 0.02^a^	2.30 ± 0.01^a^	2.22 ± 0.01^b^	ND
C15	3.62 ± 0.02^a^	3.51 ± 0.03^b^	3.63 ± 0.03^a^	3.12 ± 0.02^c^
C16	89.81 ± 0.31^d^	98.70 ± 0.33^c^	112.30 ± 0.22^a^	107.22 ± 0.12^b^
C16:1 (n7)	81.02 ± 0.21^b^	118.81 ± 0.21^a^	52.41 ± 0.13^c^	39.12 ± 0.23^d^
C17	3.12 ± 0.03^a^	3.22 ± 0.04^a^	3.01 ± 0.01^b^	2.15 ± 0.02^c^
C18	2.70 ± 0.03^a^	2.71 ± 0.01^a^	2.61 ± 0.03^a^	2.61 ± 0.01^a^
C18:1 (n9)	212.81 ± 0.53^b^	226.82 ± 0.23^b^	189.92 ± 0.21^c^	112.32 ± 0.31^d^
C18:2 (n6)	219.12 ± 0.12^b^	286.33 ± 0.21^a^	169.92 ± 0.23^c^	121.23 ± 0.13^d^
C18:3 (n6)	0.21 ± 0.02^b^	0.32 ± 0.03^a^	0.13 ± 0.03^c^	ND
C18:3 (n3)	9.22 ± 0.03^b^	10.62 ± 0.01^a^	7.10 ± 0.02^c^	5.41 ± 0.02^d^
C18:4 (n3)	4.20 ± 0.02^a^	4.22 ± 0.03^a^	4.01 ± 0.03^b^	2.25 ± 0.02^c^
C20	0.91 ± 0.02^b^	1.07 ± 0.01^a^	0.73 ± 0.01^c^	ND
C20:1 (n9)	11.92 ± 0.03^b^	12.72 ± 0.11^a^	8.11 ± 0.02^d^	5.23 ± 0.01^d^
C20:2 (n6)	2.72 ± 0.02^a^	2.71 ± 0.02^a^	2.73 ± 0.03^a^	0.31 ± 0.02^b^
C20:3 (n6)	1.41 ± 0.01^a^	1.46 ± 0.01^a^	1.33 ± 0.03^b^	ND
C20:4 (n6)	5.51 ± 0.01^b^	5.82 ± 0.03^a^	5.34 ± 0.02^c^	ND
C20:4 (n3)	2.10 ± 0.02^b^	2.21 ± 0.03^a^	1.81 ± 0.02^c^	ND
C20:5 (n3)	19.11 ± 0.23^b^	19.43 ± 0.11^a^	17.41 ± 0.11^c^	8.27 ± 0.21^d^
C22	0.41 ± 0.01^a^	0.42 ± 0.02^a^	0.20 ± 0.02^b^	ND
C22:1 (n9)	1.11 ± 0.02^b^	1.21 ± 0.01^a^	0.91 ± 0.03^c^	ND
C22:2 (n6)	4.81 ± 0.01^b^	5.10 ± 0.01^a^	3.02 ± 0.01^c^	0.21 ± 0.02^d^
C22:4 (n6)	0.22 ± 0.02^b^	0.31 ± 0.02^a^	0.22 ± 0.03^c^	ND
C22:5 (n6)	1.40 ± 0.01^b^	1.42 ± 0.01^b^	1.54 ± 0.02^a^	ND
C22:6 (n3)	23.21 ± 0.21^b^	24.93 ± 0.13^a^	20.82 ± 0.11^c^	5.21 ± 0.13^d^
C24:1 (n9)	0.12 ± 0.02^a^	0.14 ± 0.02^a^	ND	ND
Saturated fatty acids	123.12 ± 0.22^c^	135.31 ± 0.12^b^	143.72 ± 0.12^a^	134.31 ± 0.22^b^
MUFA	313.91 ± 0.33^b^	361.92 ± 0.21^a^	253.51 ± 0.21^c^	156.67 ± 0.31^d^
PUFA	286.12 ± 0.42^b^	364.61 ± 0.31^a^	235.14 ± 0.23^c^	142.89 ± 0.13^d^
Total fatty acids	723.11 ± 0.12^b^	861.82 ± 0.13^a^	632.32 ± 0.32^c^	433.87 ± 0.22^d^
**Average parameters calculated from fatty acids composition**				
Number of identified fatty acids	25	25	24	16
Average equivalent carbon number	8.52	8.52	8.52	8.66
Average carbon number	17.96	17.96	17.83	17.44
Average double bond	1.72	1.72	1.70	1.53
Sat%	16.99	15.73	22.74	31.03
Mono%	43.42	42.05	40.16	36.06
Poly%	39.59	42.22	37.09	32.91

A: Oil extracted with chloroform:methanol (2:1, v/v), B: Oil extracted with hexane.

*Oil extracted under optimal conditions: extraction temperature = 80°C, extraction time = 25 min.

**Negative control, extraction temperature = 120°C, extraction time = 5 min.

sat%: saturated fatty acid (%), mono%: MUFA (%), poly%: PUFA (%).

Values are given as mean ± SD (*n* = 3).

Different lowercase superscripts in the same row denote significant differences (*p* < 0.05). ND: not detectable.

The fingerprint region contained three peaks, i.e., (a) a CH_2_ bending vibration at 1,362 cm^−1^, (b) an out-of-plane deformation at 1,153 cm^−1^, and (c) a CH_2_ rocking vibration at 721 cm^−1^. Among these, the observed peak at 1,153 cm^−1^ (out-of-plane deformation) is indicative of the lipid content within the sample. The 1,153 cm^−1^ peak corresponds to the deformation of C–H bonds in lipid molecules, providing insights into the degree of unsaturation and the overall lipid composition. These spectral features not only offer information about lipid characteristics but also help to understand the interactions between lipids and other components, such as proteins, within the sample [49]. The oil samples recovered with chloroform:methanol (2:1, v/v) and Run 7 had lower amplitude when compared to the oil samples recovered with hexane and optimized conditions. This showed that the oil extracted with chloroform:methanol (2:1, v/v) and Run 7 had less amount of PUFAs when compared with the oil recovered with hexane and optimal conditions. These results were in agreement with the fatty acid composition of the oils.

#### 3.3.2 Fatty acid composition

Table 4 provides the fatty acid compositions of the four selected oil samples under study. The most prominent fatty acid in all four samples was linoleic acid, constituting around 22% of the total fatty acids. The next major fatty acid was oleic acid, accounting for approximately 17% of the total fatty acids. Similarly, Sathivel et al. [50] also observed a predominance of omega-6 and omega-9 fatty acids in catfish oils. The rest of the other fatty acids were in moderate or lower concentrations.

The present study also found that the total amount of PUFA was higher (49–62%) than the total amount of saturated fatty acids (*p* < 0.05). However, EPA and DHA were present in low concentrations in all samples. Similarly, Sathivel et al. [50] observed that the long-chain omega-3 fatty acids were very low (ranging from 1.8 to 2.1 mg/g) in catfish oil. The use of different extraction methods had a significant impact on the PUFA content of the extracted oil, with hexane extraction yielding the highest PUFA content, followed by chloroform:methanol (2:1, v/v), the optimized conditions, and Run 7 (*p* < 0.05). This suggests that the thermal process during wet rendering may have a negative effect on the PUFA content [51]; however, using an optimized extraction condition could retain a considerable amount of PUFA. This finding is significant for industries seeking to extract PUFA-rich oils from fish byproducts using a more environmentally friendly and cost-effective method like wet rendering. Marsol-Vall et al. [52] also demonstrated that with proper optimization, wet rendering can yield oil with a considerable amount of PUFA, making it a viable alternative to solvent-based extraction methods. Additionally, longer extraction times could degrade PUFA to a greater extent [10]. The oil obtained underoptimized conditions had significantly higher amounts of EPA and DHA (17.41 and 20.82 μg/g, respectively) compared to the oil extracted from Run 7 (8.27 μg/g EPA and 5.21 μg/g DHA, respectively), which could be due to the higher rate of oxidation in oil extracted from Run 7, leading to the production of secondary oxidation compounds. Extracting EPA and DHA from fish byproducts like catfish heads aligns with sustainable practices by minimizing waste in the fish processing industry. The higher EPA and DHA content achieved through optimized wet rendering conditions underscores the cost-efficiency of this method while maintaining nutritional value.

#### 3.3.3 Volatile compounds

Table 5 presents the volatile compounds present in oils obtained by both solvent and wet rendering methods. Among the volatile compounds, aldehydes were found to be the most abundant.

**Table 5:** Change in the volatile compounds of catfish head oils recovered using the wet rendering method as compared to the two solvent extraction methods

Volatile compounds	A	B	Optimal conditions*	Run 7**
**Aldehydes**				
Hexanal	0.16^c^	0.12^d^	0.42^b^	0.59^a^
Nonanal	0.04^c^	0.01^d^	0.12^b^	0.73^a^
2-Octenal	ND	ND	0.10^b^	0.35^a^
2,4-Heptadienal	ND	ND	0.09^b^	0.11^a^
Benzaldehyde	ND	ND	ND	0.05
2-Nonenal	ND	ND	0.06^b^	0.14^a^
2-Decenal	ND	ND	ND	0.02
10-Undecenal	ND	ND	ND	0.06
2-Dodecenal	ND	ND	ND	0.03
2,4-Decadienal	0.11^c^	0.07^d^	0.98^b^	1.48^a^
Hexadecenal	ND	ND	ND	0.22
17-Octadecenal	0.02	ND	0.19^b^	0.35^a^
**Ketones**				
3,5-Octadien-2-one	ND	ND	ND	0.06
Cyclohexene	ND	ND	ND	0.05
Pentadecane	ND	ND	ND	0.08
Hexadecane	ND	ND	ND	0.04
Heptadecane	ND	ND	0.02^b^	0.04^a^
**Others**				
Furan 2-pentyl	ND	ND	ND	0.09

A: Oil extracted with chloroform:methanol (2:1, v/v), B: oil extracted with hexane.

*Oil extracted under optimal conditions: extraction temperature = 80°C, extraction time = 25 min.

**Negative control, extraction temperature = 120°C, extraction time = 5 min.

Values are expressed as abundance (×10^6^).

Different lowercase superscripts in the same row denote significant differences (*p* < 0.05).

ND: not detectable.

Aldehydes are commonly used as indicators of lipid oxidation in various fats and foods containing oils, as they have low threshold values and contribute to unpleasant odors [41]. The major aldehydes identified in the current study were octanal, nonanal, pentanal, and hexanal, along with some minor aldehydes such as 2,4-decadienal and 17-octadecenal. Octanal is primarily produced by the oxidation of omega-3 fatty acids, particularly ALA [53], while nonanal, pentanal, hexanal, and 2,4-decadienal are mainly derived from the oxidation of linoleic acid and oleic acid [54]. The oils obtained by solvent extraction methods contained lower levels of aldehydes than those obtained through wet rendering methods, indicating that the temperature and time in the wet rendering process could affect oil oxidation. However, the oil obtained under the optimized conditions had lower levels of hexanal and 2,4-decadienal than Run 7.

In the current study, 3,5-octadien-2-one was detected only in the oil recovered from Run 7. This ketone is the most important product resulting from the lipid oxidation of omega-3 PUFA [55]. Additionally, the oil recovered from Run 7 was found to contain furan derivatives, such as furan 2-pentyl (Table 5). Furan 2-pentyl is believed to be generated from the 12-hydroperoxide of EPA and 16-hydroperoxide of DHA [56]. The oil recovered from Run 7 contained the highest number of volatile compounds, followed by the optimized condition.

## 4 Conclusion

This study successfully recovered oil from *C. magur* heads, rich in essential PUFA including oleic acid, linoleic acid, ALA, EPA, and DHA. The optimization process identified extraction time as the most critical variable, significantly impacting the oil yield and quality attributes, including acid value, FFA, PV, and TBARS. Additional analyses, such as FTIR, fatty acid profiling, and volatile compound assessment, corroborated these findings. Utilizing optimized conditions of 25 min of extraction time at a constant temperature of 80°C, we achieved an oil yield and quality comparable to conventional solvent extraction methods. This highlights the crucial role of extraction time in determining the oil yield and maintaining the desired composition and quality.

## Supplementary Material

Supplementary Figure
