# Supplementary material for: Influence of solvent-free extraction of fish oil from catfish (Clarias magur) heads using a Taguchi orthogonal array design: A qualitative and quantitative approach
Source: Open Life Sci. 2023 Nov 23;18(1):20220789. doi: 10.1515/biol-2022-0789 (PMC10668109; doi:10.1515/biol-2022-0789)
Supplement: Supplementary Figure [file biol-2022-0789-sm.pdf]

Supplementary material

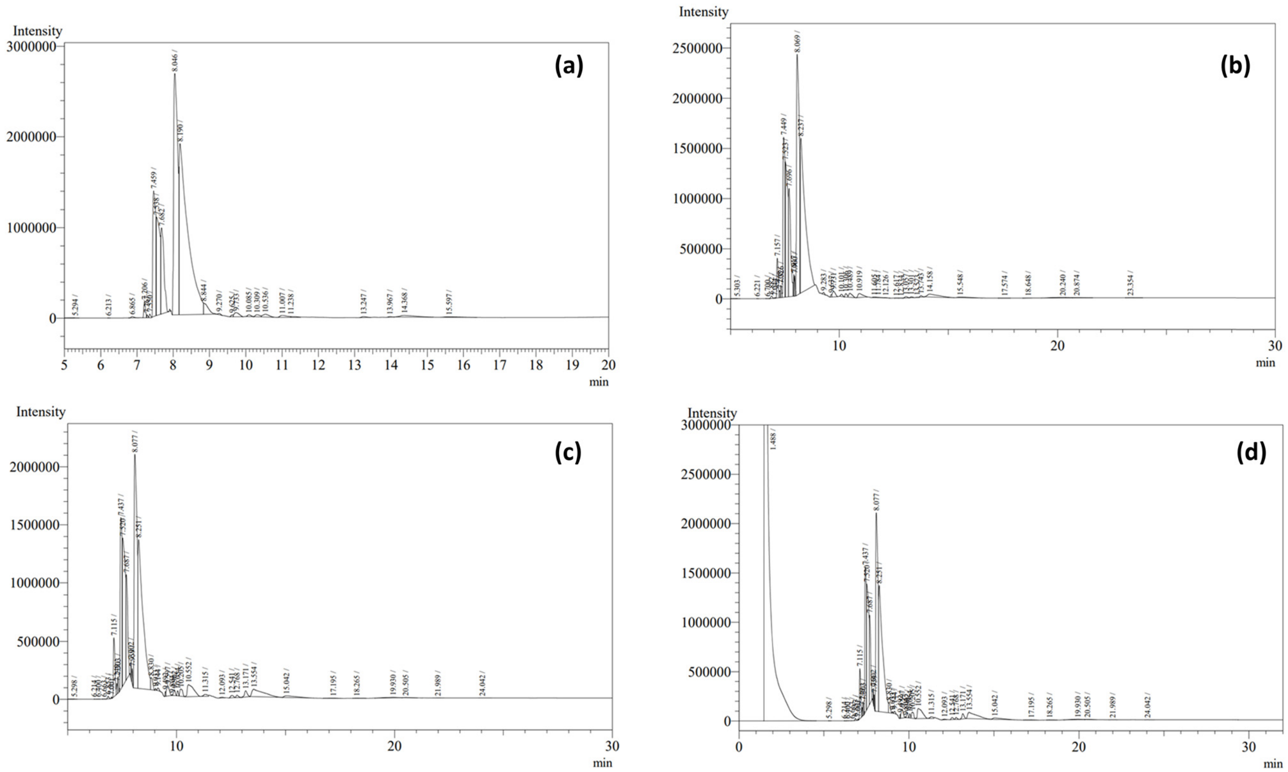

Figure S1: Chromatograms obtained from the GC-FID study of catfish head oils recovered using different methods- - A) hexane, B) chloroform: methanol (2:1, v/v), C) Optimized condition [Extraction Temperature = 80 °C, Extraction Time = 25 min], D) Run 7 [Extraction Temperature = 120 °C, Extraction Time = 5 min].

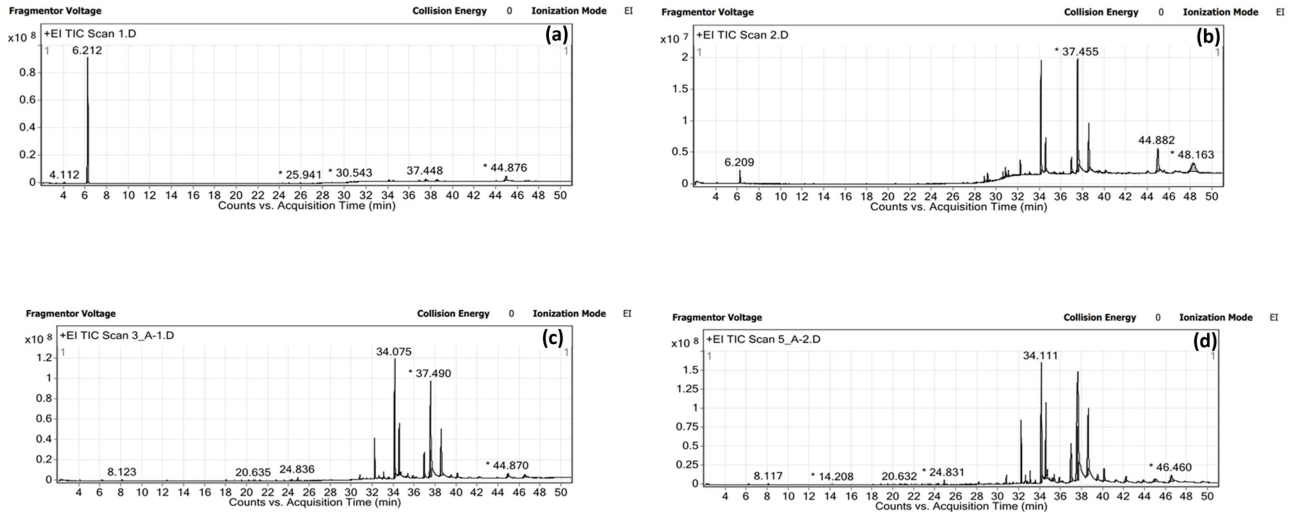

Figure S2: Chromatograms obtained from the GC-MS study of catfish head oils recovered using different methods- - A) hexane, B) chloroform: methanol (2:1, v/v), C) Optimized condition [Extraction Temperature = 80 °C, Extraction Time = 25 min], D) Run 7 [Extraction Temperature = 120 °C, Extraction Time = 5 min].

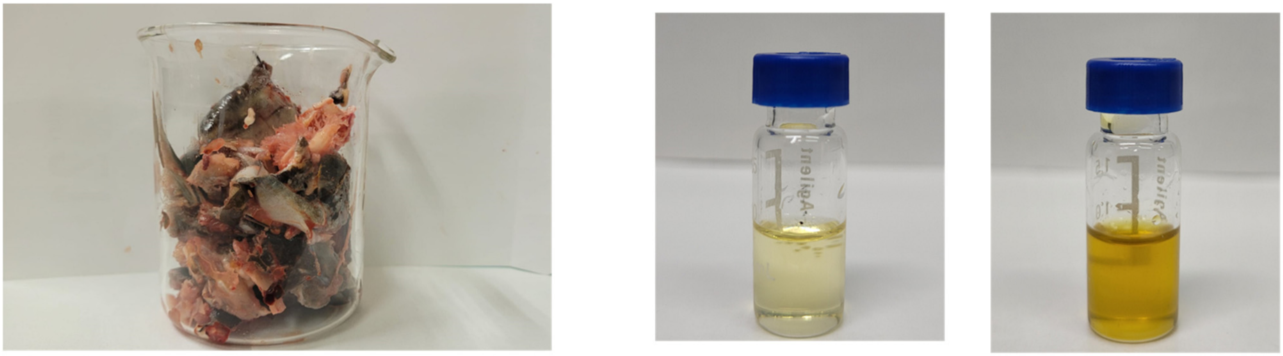

Fresh Catfish (*Clarias magur*) head (Chopped for oil extraction)

Catfish head oil (Solvent extraction method)

Catfish head oil (Optimized Wet rendering method)

Figure S3: Original images of raw materials and extracted oils.
